# Human CD38^hi^CD138^+^ Plasma Cells Can Be Generated In Vitro from CD40-Activated Switched-Memory B Lymphocytes

**DOI:** 10.1155/2014/635108

**Published:** 2014-12-23

**Authors:** Rayelle Itoua Maïga, Guillaume Bonnaure, Josiane Tremblay Rochette, Sonia Néron

**Affiliations:** ^1^Department of Biochemistry, Microbiology and Bio-Informatics, Laval University, 1045 Avenue de la Médecine, Québec, QC, Canada G1V 0A6; ^2^Hema-Quebec's Department of Research and Development, 1070 Avenue des Sciences-de-la-Vie, Québec, QC, Canada G1V 5C3

## Abstract

B lymphocyte differentiation into long-lived plasma cells is the keystone event for the production of long-term protective antibodies. CD40-CD154 and CD27-CD70 interactions are involved in human B lymphocyte differentiation into CD38^hi^CD138^+^ cells in vivo as well as in vitro. In this study, we have compared these interactions in their capacity to drive switched-memory B lymphocytes differentiation into CD38^hi^CD138^+^ plasma cells. The targeted B lymphocytes were isolated from human peripheral blood, expanded for 19 days, and then submitted to CD70 or CD154 interactions for 14 days. The expanded B lymphocytes were constitutively expressing CD39, whereas CD31's expression was noticed only following the in vitro differentiation step (day 5) and was exclusively present on the CD38^hi^ cell population. Furthermore, the generated CD38^hi^CD138^+^ cells showed a higher proportion of CD31^+^ cells than the CD38^hi^CD138^−^ cells. Besides, analyses done with human blood and bone marrow plasma cells showed that in vivo and de novo generated CD38^hi^CD138^+^ cells have a similar CD31 expression profile but are distinct according to their reduced CD39 expression level. Overall, we have evidences that in vitro generated plasma cells are heterogeneous and appear as CD39^+^ precursors to the ones present in bone marrow niches.

## 1. Introduction

T-dependent B cell activation leads to the emergence of memory B cells (B_mem_) as well as plasmablasts [1]. The former are B cells interacting with the antigen during the secondary immune response while the latter are terminally differentiating B cells. These processes occurring in lymph nodes involve numerous soluble factors and cellular interactions. Several soluble factors such as IL-2, IL-4, IL-5, IL-6, IL-21, IFN-*α*, and IFN-*γ* assure the activation and differentiation process of B cells into memory cells and plasmablasts [2–9]. Furthermore, signalling through STAT3 activation, involving IL-21 and/or IL-10 or IL-6, is considered as a critical point for the differentiation of naïve and memory B lymphocytes as well as plasma cells survival [10–12]. On the other hand, the CD40-CD154 interaction is known to be at the heart of B cell activation [13, 14]. In addition, we have shown that a very low intensity of CD154 interaction combined to a mix of IL-2, IL-4, and IL-10 can lead CD19^+^ cells [15] as well as CD27^+^IgG^+^ human B lymphocytes [16] to expand and differentiate into Ig-secreting cells. Individuals with defective CD40 fail to form germinal centers and perform isotype switching, leading to a disorder called the X-linked hyper IgM syndrome [17]. Another important cellular interaction occurs between CD27 and CD70, which are from the TNF and TNF receptor families, respectively [18]. CD27 is expressed on memory B cells while CD70 is transiently expressed on activated B, T as well as dendritic cells [19]. This interaction is known to play a key role in B cell differentiation [20–22]. Similarly to the CD40-CD154 interaction, CD70^+^ naïve cells can drive CD27^+^ memory B lymphocytes to differentiate into antibody secreting cells [13]. Sequential interactions involving CD40-CD154 and then CD27-CD70 have been proposed to drive terminal differentiation of B lymphocytes in germinal centers [23].

Following the germinal center reaction, plasmablasts can differentiate into either short or long-lived plasma cells, approximately 7 days following antigen encounter [2]. The short-lived plasma cells have a high immunoglobulin (Ig) secretion rate and die from apoptosis after a few days due to endoplasmic reticulum stress [24, 25]. As for long-lived plasma cells, they complete their terminal differentiation in survival niches found notably in the bone marrow, the spleen, lymphoid tissues, or mucosa associated lymphoid tissues (MALT) (reviewed in [26]). While these cells secrete Ig at a slower rate than short-lived plasma cells, they do so over a longer period of time, ranging from a couple of years to the life of an individual [27]. This sequence allows having an important antibody secretion at the peak of the immune response while keeping a protective antibody level in the serum several years after antigen encounter [27]. Although there is no extracellular marker allowing to distinguish between short-lived and long-lived plasma cells, they are known to highly express CD38 (CD38^hi^) and may or may not express CD138 (CD138^+/−^) [28]. Bone marrow plasma cells, which are usually considered to be long lived, are CD38^hi^CD138^+^ [29]. Due to their low concentration in the peripheral blood (2 cells/*μ*L) [28], in vivo plasma cells are hard to isolate for further characterization. Several studies aimed at generating plasma cells in vitro [15, 28, 30–35] to further understand their biology and regulation.

Given that they are at the heart of the humoral immune response [27, 36] and that they are also involved in several autoimmune diseases such as systemic lupus erythematous, monoclonal gammopathy of undetermined significance (MGUS), multiple myeloma, and macroglobulinemia as well as high chains diseases [37–41], a precise characterization of these cells is essential. Furthermore, a meta-analysis grouping the data provided by independent groups has led a study on the mRNA expression of surface markers and chemokine receptors as well as several proteins related to memory B cells, plasmablasts, and plasma cells differentiation states [35]. This large study highlighted the exclusive presence of CD31 and CD39 mRNA in bone marrow plasma cells and plasmablasts, respectively, allowing differential characterization of those two cell types.

Our team has recently reported the development of a culture system allowing large-scale expansion of switched-memory B lymphocytes [42]. Herein, we compare the capacity of CD40-CD154 and CD27-CD70 interactions to induce the differentiation of these expanded cells in vitro and characterize the generated plasma cells using two surface markers: CD31 and CD39. We have found that both interactions had the same capacity to induce plasma cell generation even though the CD27-CD70 interaction led to a faster differentiation. We have also found that these in vitro generated CD38^hi^CD138^+^ cells were different from bone marrow or peripheral blood plasma cells based on their CD39 expression. Our study suggests that such in vitro plasma cells are precursors of those found in the bone marrow and thus precursors of long-lived plasma cells.

## 2. Material and Methods

### 2.1. Human Mononuclear Cells and Memory B Lymphocytes

This study has been approved by Héma-Québec's Research Ethics Committee. Regular platelet donors who agreed to participate in this study have all signed an informed consent statement. Peripheral blood mononuclear cells (PBMNCs) were recovered from leukoreduction chambers by centrifugation on Ficoll-Paque and stored frozen as previously described [43]. CD19^+^ B lymphocytes were isolated from PBMNCs by negative selection using the EasySep B Cell Enrichment kit following manufacturer's instructions (Stem Cell Technologies, Vancouver, BC, Canada). Switched-memory B cells, representing IgG^+^, IgA^+^, and IgE^+^ cells, were further isolated using an EasySep custom cocktail, which removes most IgD^+^ and IgM^+^ cells (Stem Cell Technologies). This two-step negative selection provided untouched switched-memory B lymphocytes with a purity level exceeding 95% as determined by flow cytometry analyses [42].

### 2.2. Bone Marrow Mononuclear Cells

Bone marrow mononuclear cells were obtained from Lonza (Walkerville, Maryland, USA). Prior to assays, the cells were thawed and incubated overnight in IMDM medium supplemented with 10% ultra-low IgG fetal bovine serum (FBS) (Invitrogen, Life Technologies, Burlington, ON, Canada).

### 2.3. CD154^+^ and CD70^+^ Adherent Cell Lines

L4.5 and 3H7 cells are genetically modified L929 cell lines (CCL-1, American Type Culture Collection, Manassas, VA) expressing CD154 [44] or CD70 [45], respectively. L4.5 cells expressing about 21,000 CD154 molecules per cell and 3H7 cells expressing about 25000 CD70 molecules per cell [15] were cultured in IMDM medium supplemented with 5% FBS. For B lymphocytes cocultures, L4.5 and 3H7 cells were irradiated with 7500 rad using a GammaCell 1000 Elite ^137^Cs-*γ*-irradiator (Nordion International, Kanata, Canada).

### 2.4. Culture of Human Switched-Memory B Lymphocytes

All cultures were done in IMDM supplemented with 10% ultra-low IgG FBS containing 10 *μ*g/mL insulin, 5.5 *μ*g/mL transferrin, and 6.7 ng/mL sodium selenite (all from Invitrogen). Purified switched-memory B lymphocytes were submitted to a three-step culture protocol. Firstly, the cells were seeded at 3 × 10^5^ cells/mL in 6-well Primaria plates (BD Biosciences, Mississauga, ON, Canada) with 1.8 × 10^5^ *γ*-irradiated CD154^+^ L4.5 cells corresponding to about 5 B cells for 1 L4.5 cell [15, 44]. This ratio is referred in the study as the high interaction level. The medium was supplemented with 5 ng/mL IL-2 (≥50 U/mL), 40 ng/mL IL-10 (≥20 U/mL) (both from PeproTech, Rocky Hill, NJ, USA), and 3.5 ng/mL IL-4 (≥100 U/mL) (R&D Systems, Minneapolis, MN, USA) [42]. When indicated, an expanded-memory-B-cell bank at day 19 was cryopreserved and thawed when needed for further differentiation assays. Secondly, thawed expanded cells were cultured using a lower CD154 : CD40 (or CD70 : CD27) ratio corresponding to one L4.5 (CD154^+^) or 3H7 (CD70^+^) cell per 20 B cells (1 : 20 ratio). This ratio is referred in the study as the low interaction level. To initiate differentiation, a transition step was performed by adding 12.5 ng/mL IL-6 (≥125 U/mL, Peprotech) to the IL-2, IL-4, and IL-10 mixture. The third phase started 4 to 5 days later. The 1 : 20 ratio was maintained and the medium was only supplemented with IL-6 (12.5 ng/mL) and IL-10 (40 ng/mL) until the end of the cultures. Refer to the supplementary material (see Figure S1 in the Supplementary Material available online at http://dx.doi.org/10.1155/2014/635108) for the experimental model schematic. When indicated, B lymphocytes were washed between the transition and the differentiation phases to remove residual cytokines. In addition, a combination of L4.5 cells and 3H7 cells was used in equal part to constitute a high and low ratio. Of note, L4.5 [44] and 3H7 [20] cell lines are genetically modified L929 cell line. Consequently, the L929^mock^ cell line was used as a negative control during the differentiation phase. Cultures were fed by adding fresh medium every 2-3 days and *γ*-irradiated L4.5 or 3H7 cells were renewed every 4-5 days, maintaining the low level of CD40-CD154 interaction. Cell counts and viability were evaluated in triplicates by Trypan Blue exclusion using a hemocytometer.

### 2.5. Flow Cytometry Analysis

FITC-conjugated anti-CD31, PE-conjugated anti-CD38, and PCP-efluor 710-conjugated anti-CD39 were all from eBioscience (San Diego, CA, USA). Cells were also stained with PE-Cy7-conjugated anti-CD19 (BD Pharmingen, Mississauga, ON, Canada), PCP-Cy5.5-conjugated anti-CD38, V500-conjugated anti-CD45, alexa fluor 488-conjugated anti-CD20, APC-conjugated anti-CD27 (BD Biosciences), krome orange conjugated-CD45 (Immunotech, Mississauga, ON, Canada), alexa efluor 647 or PE-conjugated anti-CD138 (BA38) (AbD Serotec, Raleigh, NC, USA), APC-conjugated anti-CD39, and PE-Cy7-conjugated anti-CD38 (Biolegend, San Diego, CA, USA). Dead cells were discarded using Pacific Blue and Sytox Blue staining (Molecular Probes, Life Technologies, Burlington, ON, Canada) or 7-aminoactinomycin D (BD Biosciences). Cells stained with Pacific Blue were fixed with 2% formaldehyde. Data acquisition was done immediately or 18 to 20 hours after staining with a CyFlow ML flow cytometer and the FlowMax 3.0 software (Partec, Münster, Germany) by gating on 10 000 to 300 000 events. Data was subsequently analyzed with FCS Express 4 software (De Novo Software, Los Angeles, CA, USA). For all analyses, cells were selected based on a SSC/FSC plot after which live cells were selected for further analyses of bone marrow or blood mononuclear cells as well as cultured cells according to CD45 expression. Quadrants were determined using fluorescence minus one (FMO) controls.

### 2.6. Fluorescence Microscopy

Differentiated cells morphology was visualized by fluorescence microscopy. Nuclei were stained with DAPI (Sigma Aldrich, ON, Canada) and F-actin was labeled with phalloidin 594 (Molecular Probes, Life technologies). Briefly, cells were concentrated on microscope slides using the Cytospin 4 Cytocentrifuge (Thermo Scientific, Rockford, Illinois, USA) and spin at 500 rpm for 5 minutes. They were fixed with 2% paraformaldehyde, at room temperature, for 30 minutes, after which they were incubated during 20 minutes with a 2% BSA-0.01% saponin solution. Actin labeling was done by incubating the cells with a 1 : 40 phalloidin solution for an hour. They were then washed twice with a 0.1% NP-40 solution and incubated with a 100 ng/mL DAPI solution and mounted with Prolong Gold antifade reagent (Invitrogen, Life Technologies) and a cover slip. Images were captured using a Nikon Eclipse TE 2000-S microscope (Nikon, Mississauga, ON, Canada) with 100x plan immersion oil objective and TsView 7 software (Xintu Photonics Co., Ltd, Fuzhou, China). They were merged using ImageJ software (National Institutes of Health, Bethesda, MD, USA).

### 2.7. Determination of Ig Concentrations

For the determination of IgG and IgM secretion rates, cells were harvested at indicated days, washed with PBS-glucose, and seeded at 1-2 × 10^6^ cells/mL in IMDM medium supplemented with 10% FBS. Supernatants were collected after 6 hours and IgG and IgM concentrations were determined by ELISA as previously described [46]. In addition at indicated days, IgA and IgG contents were also determined by ELISA directly in the supernatants. IgA, IgE, and IgG subclasses and IgM levels were quantified on days 5 and 14 in culture supernatants using the Bio-Plex Pro Human Isotyping kit (Bio-Rad, Hercules, CA, USA). The assay was carried out in a flat-bottom Immulon-II plate and the plate was washed by hand on a 96-well magnet. The rest of the assay was performed following the manufacturer's instructions and the plate was read on a Luminex IS 100 Instrument (Luminex, Austin, Texas, USA).

### 2.8. Statistical Analyses

The appropriate post hoc tests used are described in the results section.

## 3. Results

### 3.1. Determination of the Best Cytokine-Environment for B Cell Differentiation

**Figure 1:**
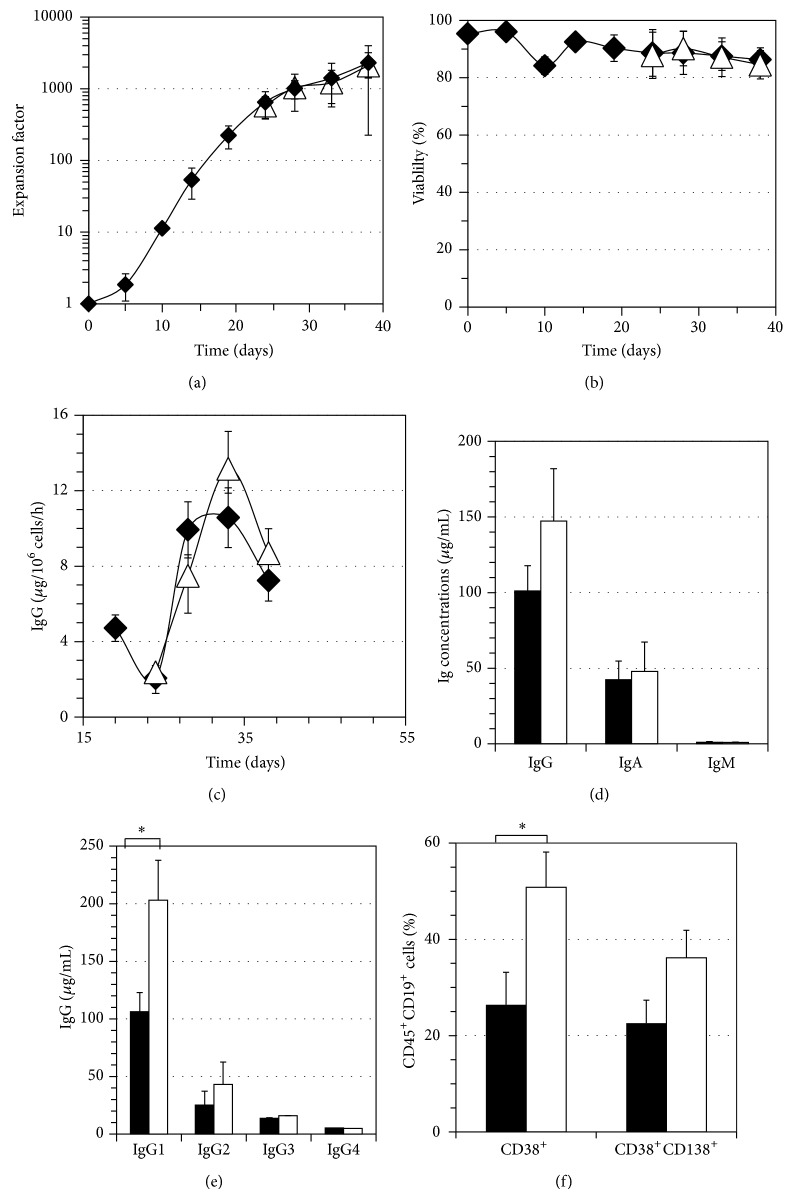
In vitro plasma cell generation and cytokine microenvironment. B lymphocytes were expanded for 19 days with IL-2, IL-4, and IL-10 at high interaction level with CD154. The same interleukin combination was used for the differentiation phase (filled symbols and bars) or the cells were cultured from day 19 with a combination of IL-6 and IL-10 (empty symbols and bars). The transition and differentiation phases lasted a total of 18 days with a low CD154 interaction. The results shown are the mean of 6 independent experiments. (a) Cell expansion, (b) cell viability, (c) IgG secretion rate in the differentiation environment, from D19 to D38, (d) IgG, A, and M secretion on day 33 supernatants, and (e) IgG subclasses secretion on day 33 supernatants. A significant difference was noticed in IgG1 secretion among the two interleukins combinations. Statistical analyses were done using the Bonferroni *t*-test and the *P* value was <0.05 (f) CD38 and CD38^+^CD138^+^ cells frequency. The IL-6-10 combination generated a larger CD38^+^ cell population on day 33, confirmed by a paired *t*-test, *P* value = 0.0188. All errors bars stand for SD and can be smaller than symbols.

The combination of IL-2, IL-4, and IL-10 along with high CD40-CD154 interaction has been proven to stimulate long-term memory B cell expansion in vitro [42]. Based on the fact that a lower CD40-CD154 interaction leads to B cell differentiation [15], we have compared the IL-2, IL-4, and IL-10 cytokine combination to that of IL-6 and IL-10 in promoting in vitro differentiation at a low CD40-CD154 interaction. This comparison was based on the capacity of either combination to induce immunoglobulin secretion and emergence of CD38^+^ or CD38^hi^CD138^+^ cells. Cells were expanded for 19 days in a medium containing IL-2, IL-4, and IL-10 [16, 42] followed by a 4-day-long transition phase and a 18-day differentiation phase with IL-2-4-10 or IL-6-10 combinations. As shown in Figure 1(a), the passage to the differentiation environment in both conditions slowed cell expansion (Figure 1(a)) and allowed to maintain cell viability above 80% (Figure 1(b)). Being an important feature of B cell differentiation, immunoglobulins secretion was measured in both conditions. IgG secretion rate reached up to 11 ± 2 *μ*g/10^6^ cells/h with the IL-2-4-10 combination and 13 ± 2 *μ*g/10^6^ cells/h with the IL-6-10 combination on day 33 (Figure 1(c)). Differentiated cells also showed an important IgA secretion in supernatants, reaching 43 ± 12 *μ*g/mL for the IL-2-4-10 combination and 48 ± 19 *μ*g/mL for the IL-6-10 combination, while having as expected a negligible IgM secretion in both conditions (Figure 1(d)). On day 38, cells cultured with the IL-6-10 combination had an IgG_1_ secretion of 203 ± 157 *μ*g/mL, which was almost twofold higher than the 106 ± 65 *μ*g/mL measured for the IL-2-4-10 condition (Bonferroni *t*-test *P* < 0.05) (Figure 1(e)). As for phenotypic cell differentiation, we noticed a more important CD38^+^ cell population with the IL-6-10 combination, reaching up to 51 ± 7% of all CD45^+^CD19^+^ cells by day 33. Both interleukins combinations led to the emergence of CD38^+^CD138^+^ plasma cells, 23 ± 4% for IL-2-4-10, and 38 ± 4% for IL-6-10 (Figure 1(f)). Overall, the IL-6-10 combination enhanced IgG_1_ secretion and generated a higher frequency of CD38^+^ cells indicating that this cytokine combination was more favorable to the in vitro generation of plasma cells.

Besides, we have also evaluated the capacity of IL-6, IL-21, and IFN-*α*, used alone, in pairs or combined, in an attempt to improve differentiation of switched-memory B lymphocytes into plasma cells. None of these soluble factors enhanced plasma cell maturation or viability more than the combination of IL-6 and IL-10 (data not shown).

### 3.2. CD27-CD70 Interaction Leads to a Faster Differentiation

**Figure 2:**
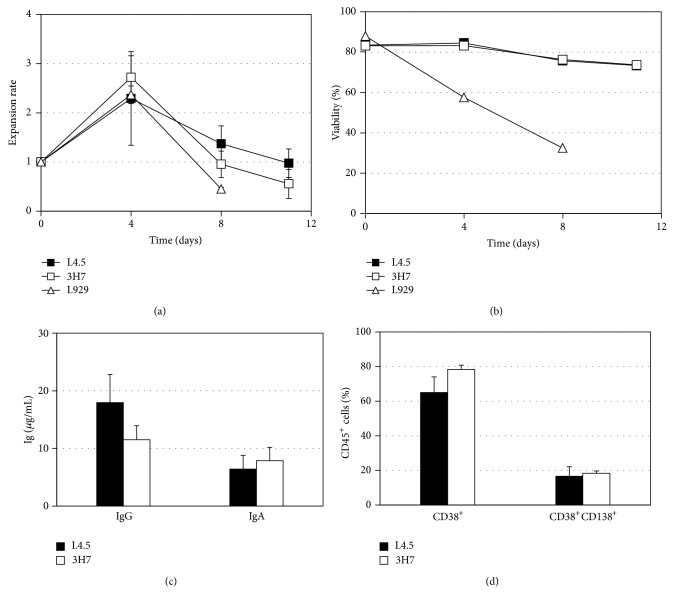
Proliferation and viability following low CD70 and CD154 interaction. B lymphocytes were monitored following their stimulation with CD70 and CD154 as well as for their response to the mock L929 cell line using a low ratio (1 : 20) for all support cells. (a) Proliferation and (b) viability were measured on days 4, 8, and 11. The time laps between days 0 and 4 are the transition phase and differentiation phase was started on day 4 and lasted until day 11. (c) Secretion of IgG and IgA was measured by ELISA assay with the pooled supernatant harvested on days 6, 8, and 11. (d) At the end of the differentiation phase (day 11), B lymphocytes were analyzed using flow cytometry to evaluate the frequency of CD38^+^ cells and CD38^+^CD138^+^ cells. These results are representative of 5 independent experiments. All errors bars stand for SD and can be smaller than symbols.

**Figure 3:**
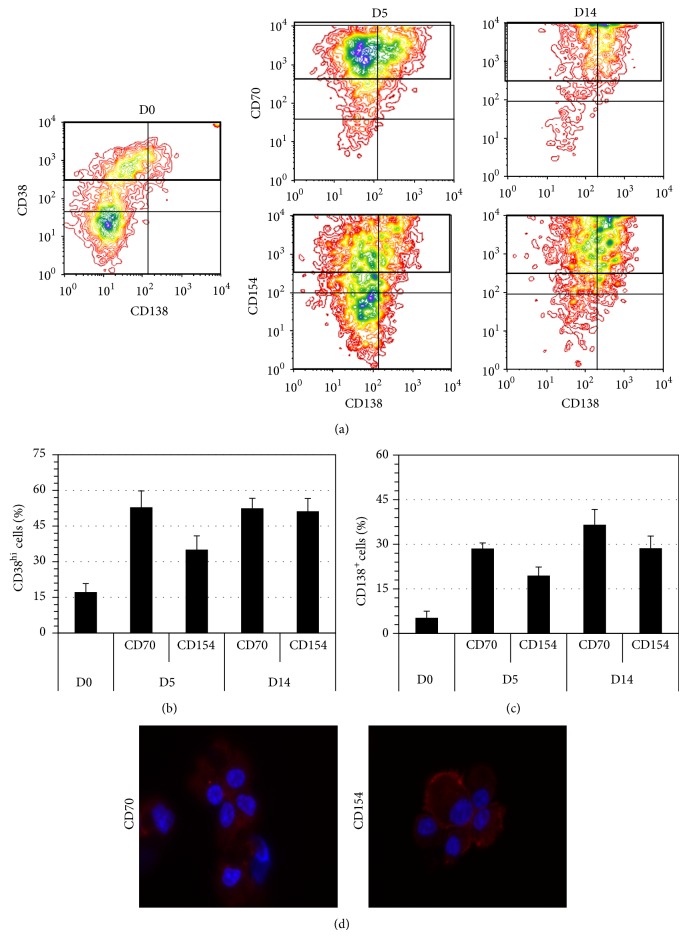
Generation of plasma cells from switched-memory B lymphocytes. Monitoring of plasma cell generation in the differentiation phase was done by flow cytometry and all analyses were done on viable cell populations. The results are presented as the mean ± S.E.M. of 6 independent samples: (a) CD38 and CD138 expression profiles on D0, 5 and 14. D0 being the day that the expanded memory B cells were thawed and were put in culture with CD70^+^ or CD154^+^ cell lines. (b) CD38^hi^ cells frequency. The comparison of CD38^hi^ cells frequency at D5 was determined using a Mann-Whitney test, *P* value = 0.0260. For the monitoring of CD138^+^ cells frequency (c), the Kruskal-Wallis test was used and no significant difference was observed. (d) Generated plasma cells morphology at D14 shown by fluorescence microscopy (100x immersion oil objective). Blue: nucleus; red: actin. Representative cells are shown.

To verify whether CD40-CD154 and CD27-CD70 interactions were similarly competent for in vitro differentiation of switched-memory B lymphocytes, we used a cryopreserved expanded-memory B lymphocytes bank. In all following experiments, the day 0 (D0) is the first day the thawed cells were put in culture to induce their differentiation. For the differentiation phase in the presence of IL-6 and IL-10, adherent cell lines expressing CD70 (3H7) or CD154 (L4.5) were used to generate a low level of interaction stimulation (Figures 2 and 3).

The level of proliferation was thus compared for both stimulation and a negative control was performed using the cell line L929^mock^ at a ratio of 1 for 20 B lymphocytes. During the transition from day 0 to day 4, the switched-memory B lymphocytes were able to expand, by 2-fold to 3-fold in all conditions (Figure 2(a)). However, the L929^mock^ cells were unable to sustain viability of the B cells, which was decreasing rapidly from day 4 (60% ± 4%) to day 8 (32% ± 2%) (Figure 2(b)). The B lymphocytes submitted to CD154 or CD70 low level of interaction stopped proliferating on day 8 while their viability was maintained until day 11 at 73 ± 2% and 73 ± 3%, respectively (Figures 2(a) and 2(b)). In addition, flow cytometry analyses using 7-AAD showed that at that time point, the viability of CD154 and CD70 stimulated cells was about 82 ± 2% and 84 ± 3% in these two conditions, respectively. Moreover, monitoring of cell division using CellVue Jade assay allowed us to notice an important decrease of cell division when cells where put in the in vitro differentiation environment compared to the cells in the expansion phase. Between days 8 and 11 in the differentiation phase (including the transition), we observed an absence of cell division while 3 to 4 divisions were observed in conditions with a high level of interaction of CD27-CD70 or CD40-CD154 (data not shown).

Finally, CD154 interaction slightly increased IgG secretion compared to CD70 interaction (Figure 2(c)). However IgA secretion was quite similar between the two conditions. The frequency of CD38^+^ and CD38^+^CD138^+^ cells was also similar in both conditions. In those cultures, the differentiation in the presence of CD154 and CD70 resulted, respectively, in a frequency of 65 ± 7% and 43 ± 7% CD27^+^ cells inside the CD138^+^ population. In regard to CD20 expression, only 2, 9 ± 0, 7% and 2, 6 ± 0, 6% of cells were positive to this marker at the end of the differentiation phase (data not shown).

We then compared the differentiation of B lymphocytes during a 14-day period in the presence of IL-6-10 combination. Figures 3(a), 3(b), and 3(c) show the evolution of the expression of CD38 and CD138 on cultured cells in both conditions. At D0, nearly half the cells were expressing CD38 and the proportion of CD38^hi^ cells was of 17 ± 4%. Five days after the cells were placed in a differentiation environment with either CD70 or CD154 cellular interactions, a higher proportion of CD38^hi^ cells was generated with the CD70 interaction (53 ± 7%) than with the CD154 interaction (35 ± 6%) (*P* = 0.0260, *n* = 6; Mann-Whitney). By day 14, the proportion of differentiated cells in both conditions reached 50%. Increased frequency of CD138^+^ cells, which was however not significant (Bonferroni multiple comparison test, *P* = 0.0564), was also noted on days 5 and 14 in the presence of CD70, 28% ± 7%, and 36% ± 12% respectively, compared to 19% ± 7% and 27% ± 13% with the CD154 interaction. Additionally, both interactions led to a significant increase in the proportion of CD38^hi^ and CD138^+^ generated cells when compared to the initial profiles, namely, at the end of the expansion phase (Figures 3(b) and 3(c)). During this 14-day differentiation phase, the total expansion was negligible, that is, less than 2-fold in both conditions, and the viability was around 80% on day 5 and decreased gradually to approximately 50% on day 14 (data not shown).

Immunofluorescence labeling confirmed the plasma cell-like morphology of the generated cells (Figure 3(d)). The staining highlighted the elliptical cell shape, shown through actin labeling in red and the very large cytoplasm with a decentered nucleus, which are typical plasma cell features. These observations were made on cells generated with CD154 or CD70 cellular interactions.

### 3.3. Important Immunoglobulin Secretion by In Vitro Differentiated B Cells

**Figure 4:**
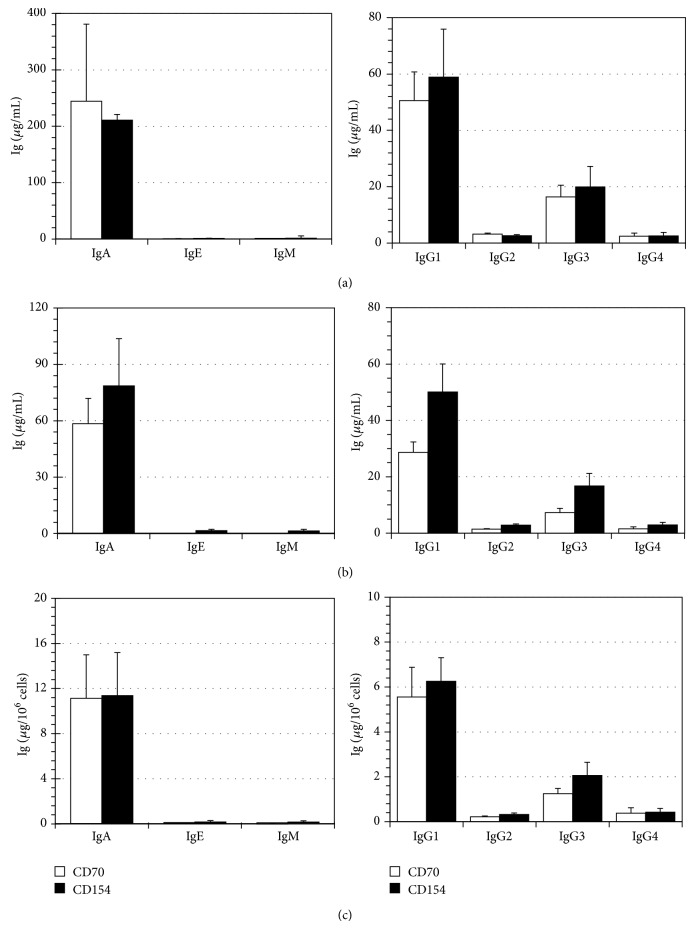
In vitro generated plasma cells secrete high level of IgA and IgG. Immunoglobulin secretion in supernatants was measured by the Bio-Plex human isotyping kit. The cumulated Ig concentration was determined on (a) day 5 (D5) after the transition phase and (b) day 14 (D14) at the end of differentiation. (c) Relative secretion was determined on D14 by normalizing immunoglobulin concentrations with total cell count on day 5, corresponding to the plateau in proliferation. The results are presented as the mean ± S.E.M. of 6 independent samples. No differences were observed between CD70 and CD154 conditions.

To further compare the B cells generated by each interaction, immunoglobulins concentration was determined in culture supernatants on days 5 (Figure 4(a)) and 14 (Figure 4(b)). As shown, five days into the differentiation environment led to equivalent IgA and IgG secretion from cells in both interaction settings. IgG_1_ was as expected, according to human serum level, the most secreted IgG subclass with a concentration of 51 ± 10 *μ*g/mL following CD70 interaction and 59 ± 17 *μ*g/mL following CD154 interaction. IgA was secreted at a very high level, with 244 ± 137 *μ*g/mL following CD70 interaction and 211 ± 112 *μ*g/mL following CD154 interaction. However in contrast to IgG_1_, IgA secretion was highly variable among samples, ranging from 36 to 915 *μ*g/mL for CD70 interaction on day 5. IgG_1_ concentration was maintained for cells cultured with CD154 until day 14 (51 ± 10 *μ*g/mL), while cells cultured with CD70 showed a decreased secretion of that immunoglobulin subclass (29 ± 4 *μ*g/mL). Moreover, IgG3 secretion with CD154 interaction (17 ± 4 *μ*g/mL) was also twofold higher than with the CD70 interaction (7 ± 2 *μ*g/mL). Based on the high viability and absence of proliferation during the transition phase (D0 to D5), a normalization according to cell concentrations on day 5 was done in order to compare secretion levels on day 14 (Figure 4(c)). Overall, all immunoglobulin secretion rates were equivalent in both conditions indicating similar differentiation status of generated plasmablasts and/or plasma cells.

The pattern of IgG secretion was also determined by a standard ELISPOT assay (see Supplementary Material, Figure S2) for B lymphocytes submitted to CD70 or CD154 in differentiation conditions and was compared to that of plasma cells present in bone marrow samples. Differentiating B lymphocytes showed a heterogeneous IgG secretion pattern composed of small and large spots, suggesting the presence of different subtype of terminally differentiated IgG secreting cells.

### 3.4. CD31 and CD39 Expression on Expanded B Cells

**Figure 5:**
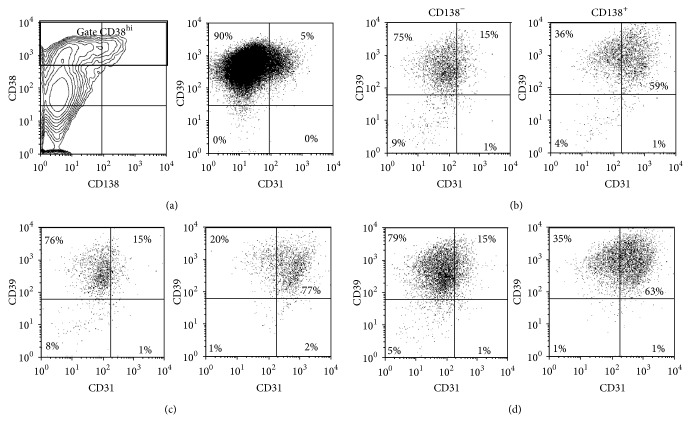
High frequency of CD39^+^ cells among the expanded B lymphocyte population. Viable cells were analyzed for CD31 and CD39 expression before their transfer into differentiation conditions (D0). (a) Profiles of total viable cells according to CD38 and CD138 (left) and to CD31 and CD39 (right) are shown. The CD38^hi^CD138^+/−^ cells represented 22 ± 3% of the cell population in the presence of CD154 interaction (b) and 25 ± 6% in the presence of CD70 (c). They represented 14 ± 2% of the population when being in the simultaneous presence of both interactions (d). These cells were further analyzed for their expression of CD31 and CD39 according to their expression of CD138 as indicated in (b, c, and d). These results are representative of 11 and 10 independent experiments for the stimulation with L4.5 cells (b) or 3H7 cells (c), respectively. The results in (d) are representative of three independent experiments.

To further characterize the emergent CD38^hi^ cells, we analyzed their phenotypes according to CD31 and CD39 expression before their transfer into the differentiation conditions. As shown previously (Figure 3(a)), about half (52 ± 5%, *n* = 8) of the expanded cells were positive for CD38 expression and less than 10% were CD138^+^ (6 ± 3%, *n* = 8) at D0. Moreover, 22 ± 3% of the expanded cells highly expressed CD38 and up to 95 ± 4% of them were CD39^+^ with a MFI of 592 ± 63 (Figure 5(a)). This result was observed independently of their CD138 expression. At this step, only a small proportion of cells were CD31^+^ (9 ± 3%). By the end of the differentiation phase, this cell population reached 35 ± 2% following CD154 interaction and 44 ± 7% following the CD70 interaction. Furthermore, analyses done on purified B cells before they were expanded showed that 85 ± 3% (*n* = 3) were positive for CD39 expression (MFI = 238 ± 15) (data not shown). Therefore, the frequency of CD39^+^ cells remained constant during in vitro cell expansion (data not shown).

### 3.5. Distinct Plasma Cell Populations In Vitro

**Figure 6:**
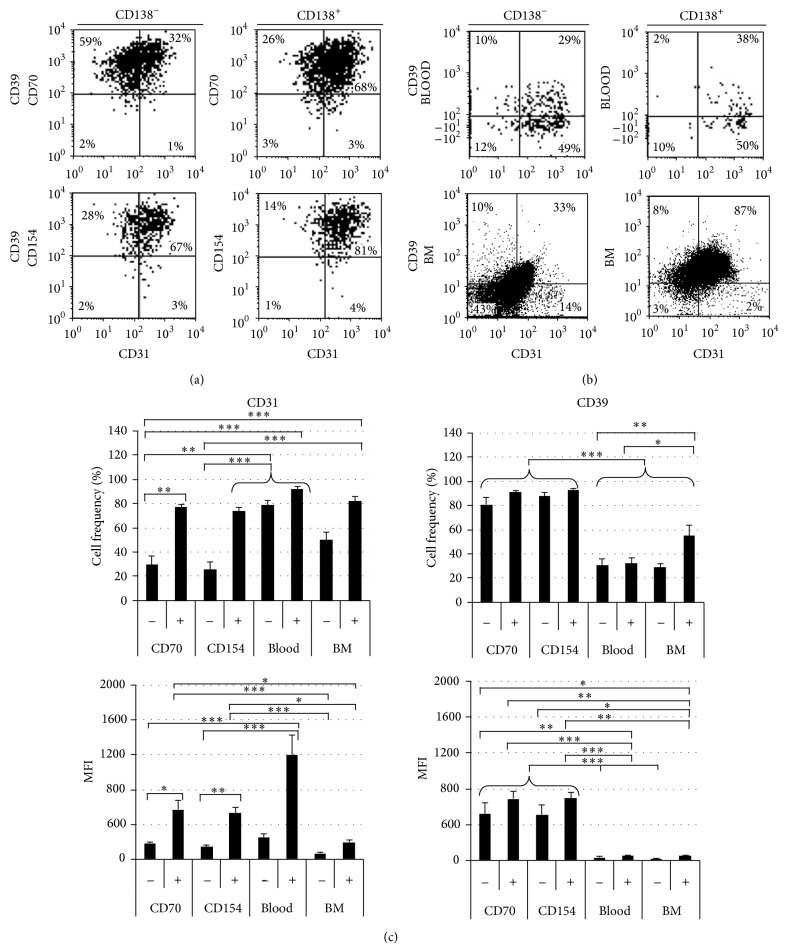
CD31 as an in vivo and in vitro generated plasma cell marker. Analyses were done on cells submitted to 14 days in differentiation conditions. (a) D14 viable CD38^hi^ cells were analyzed based on their CD31 and CD39 expression. Plots are representative of 10 independent experiments for the CD70 interaction and 13 for the CD154 interaction. (b) Bone marrow and peripheral blood plasma cells (viable CD45^+^CD38^hi^). Plots are representative of 10 and 14 independent experiments for bone marrows and for peripheral blood, respectively. (c) Comparison of CD31 and CD39 expression and frequency on generated (CD70 and CD154) and in vivo (PB and BM) plasma cells. CD138^−^ (−) and CD138^+^ (+) plasma cells expression profiles were compared using the Unpaired *t*- or the Mann-Whitney test (^*^indicating a significant difference with *P* values < 0.03). The expression profile of CD138^−^ (−) cells from different origins was compared (^**^indicating significant differences with *P* values < 0.02) as well as CD138^+^ (+) plasma cells (^***^indicating significant differences with *P* values < 0.006) using the Kruskal-Wallis test.

We then investigated how CD39 and CD31 evolved on differentiated CD38^hi^CD138^+/−^ cells after 11 or 14 days in an in vitro differentiation environment. As observed for expanded B cells (Figure 5(b)), more than 90% of generated plasma cells were positive for CD39, regardless of the interaction with CD70 or CD154 (Figure 6(a) and Table 1). Moreover, culture conditions did not seem to influence the frequency of CD31^+^ cells as CD70 and CD154 interactions led to the emergence of, respectively, 76 ± 2% and 74 ± 3% CD31^+^ cells among the CD38^hi^CD138^+^ cells (Figure 6(c) and Table 1). However, a smaller proportion of the CD38^hi^CD138^−^ cells expressed CD31. Following the CD70 or CD154 interactions, only 37 ± 7% and 25 ± 7%, respectively, expressed CD31. This difference was also observed through the CD31 mean fluorescence intensity (MFI) that was much higher for CD38^hi^CD138^+^ cells (MFI 568 ± 108) than for CD38^hi^CD138^−^ cells (180 ± 19) when submitted to CD70 interactions. Overall, four populations of CD38^hi^CD39^+^ were observed in vitro following CD70 or CD154 interactions, namely, CD138^+^CD31^+^, CD138^+^CD31^−^, CD138^−^CD31^+^, and CD138^−^CD31^−^ cells.

### 3.6. CD31 and CD39 Expression on Bone Marrow and Peripheral Blood Plasma Cells

The frequency of CD38^hi^CD138^−^ and CD38^hi^CD138^+^ was evaluated in 14 peripheral blood (PB) samples and ten bone marrow (BM) samples (Table 1). In PB, about 0.3% of CD38^hi^ cells were detected, which can be separated into equal frequency of CD138^−^ and CD138^+^ cells as reported elsewhere [28]. In BM, we observed about 2% of CD38^hi^ among the mononuclear cells [47], which included about 25% of CD138^+^ cells. The phenotype of PB and BM CD38^hi^ plasma cells was then evaluated according to CD31 and CD39 (Figure 6(b) and Table 1). PB plasma cells were predominantly CD31^+^, with 81 ± 5% of CD138^−^ cells and 93 ± 2% of CD138^+^ cells. Furthermore, CD31 expression was fivefold higher on CD138^+^ plasma cells (MFI = 1547 ± 223) versus CD138^−^ plasma cells (MFI = 310 ± 44). In the blood, CD39^+^ cells frequency within the CD38^hi^CD138^+/−^ population was lower (34 ± 5%) than their in vitro generated counterpart. BM plasma cells showed a significant difference of CD39^+^ cells frequency, with 33 ± 2% of CD38^hi^CD138^−^ and 55 ± 2% of CD38^hi^CD138^+^ cells. Besides, the proportions of CD31^+^ cells were, respectively, of 43 ± 2% and 80 ± 3% among those plasma cell subsets. Though the proportions of CD39^+^ and CD31^+^ plasma cells were similar in the PB and BM, the expression level of CD31 was eight times higher on PB CD138^+^ plasma cells compared to the same population in the BM (MFI: 194 ± 12).

Overall, there was no significant difference in the proportion of CD31^+^CD138^+^ plasma cells found in vivo and in vitro. There was however a striking difference in CD39 expression. Indeed, the expression level of this surface marker was more than 10 times higher on vitro generated CD138^+^ plasma cells than PB and BM plasma cells.

## 4. Discussion

In this study, we have shown that CD27-CD70 and CD40-CD154 interactions can both induce switched-memory B lymphocytes differentiation into CD38^hi^CD138^+/−^ plasma cells. We also demonstrated that such in vitro generated plasma cells were heterogeneous according to their CD31 and CD39 expression profiles. We observed that the final frequency of generated CD38^hi^CD138^±^ cells was equivalent in both conditions after 14 days. However, we also found that the CD70 interaction led to a much faster differentiation in the first 5 days. The functional potential of the newly generated plasma cells with both interactions was assessed and found to be similar with IgA and IgG_1_ secretion as high as 200 *μ*g/mL and 60 *μ*g/mL, respectively. Further characterization of the in vitro generated CD38^hi^CD138^+/−^ plasma cells also showed that CD39 was present on more than 80% of these cells while CD31 was present on about a quarter of the CD138^−^ and on approximately 75% of the CD138^+^ population with a significantly higher frequency as well as level of expression on the latter plasma cell subset.

The selected in vitro differentiation environment was based on low levels of CD70 and CD154 interactions combined to IL-6 and IL-10. These conditions were found to be the most effective for commitment to differentiation with concomitant negligible proliferation. It has been largely reported that IL-6 and IL-10 were excellent to drive B cell differentiation drivers [5, 48]. Moreover, in accordance with the reported in vivo process in germinal centers, the switch from expansion to differentiation led to a reduced cell expansion rate in our culture system [1]. The fast differentiation induced by the CD70 interaction between day 0 and 5 can illustrate the rapid in vivo differentiation of memory B lymphocytes following antigen encounter, when CD27^+^ B cells interact with CD70^+^ T cells [23, 49], following proliferation signalling through the CD40-CD154 interaction [23]. Interestingly, the low level of CD40-CD154 interaction seems to have a delayed but similar role during long-term culture. The culture system developed in this study simulates the germinal center reaction during a secondary immune response and could be used for the production of large amounts of plasma cells in vitro. For instance, we have previously established that memory B cells can be expanded to 2000-fold in 20 days [42]. We show here that their transition into differentiation provides after 5–7 days at least 50% of CD138+ cells with a viability of 80%. Consequently, we could generate, starting with 1 × 10^6^ memory cells, nearly 800 × 10^6^ cells committed to terminal differentiation into plasma cells.

Furthermore, IgG secretion rate in this system can reach up to 13 *μ*g/10^6^ cells/h with the CD154 interaction (Figure 1), which is higher than several in vitro models developed to date [15, 30–33]. Taken together, we can conclude that the observed effects of those two interactions are due to the molecular pathway of the expressed surface molecule (CD70 on 3H7 cells and CD154 on L4.5 cells) during cellular interaction and not related to the cocultured cell line since L929^mock^, which was used to construct 3H7 and L4.5 cell lines [20, 44] did not allow B lymphocyte differentiation (Figure 2(b)). Furthermore, their use in our culture system actually highly hindered cell viability.

This model enables us to circumvent the plasma cells rarity in circulation or in the bone marrow; thus we studied two extracellular markers CD31 and CD39 in order to discriminate between plasmablasts and plasma cells, as proposed by De Vos and coll. [35]. CD31 is an adhesion molecule that participates in cellular interactions [50, 51] and is a ligand for CD38 [52]. Their interaction has been associated with the microenvironment of leukemia cells [53–55] and high CD31 density has been associated to B cell chronic lymphocytic leukemia [56]. On the other hand, CD39 is an integral membrane protein, whose main role is to metabolize extracellular ATP and ADP into AMP [57]. The CD31^+^CD39^+/−^ profile is expected for terminally differentiated B lymphocytes from peripheral blood as well as bone marrow plasma cell [29, 35, 58]. Our results show a prevalence of CD31^+^ cells (74% to 77%) among the CD38^hi^CD138^+^ population, and a lower expression (25% to 29%) within the CD38^hi^CD138^−^ in vitro generated plasma cells population. However, the expression of CD39 indicates that the in vitro generated PCs are one step preceding fully differentiated PCs and can be lost in response to migratory stimuli or the modifications of their microenvironment. Our finding that in vitro generated PCs were characterized by a higher CD31^+^ cell population among CD138^+^ plasma cells indicates that we produced a mature plasma cell population [28, 33, 59]. Moreover, increased expression of CD31 on plasma cells correlates with the mRNA expression profile in plasmablasts and plasma cells [35]. The investigation of CD31 and CD39 expression can be a useful tool for plasma cell characterization and may allow uncovering subsets with specific roles during an immune response as recently reported [60, 61]. Furthermore, the coexpression of CD31 and its ligand CD38, on plasma cells [62] suggests a role for this interaction in the plasma cell survival niche as already proposed for leukemia cells [52, 55].

In conclusion, we have developed a culture system using either CD70 or CD154 interactions enabling the in vitro culture of CD31^+^CD38^hi^CD39^+^CD138^+^ cells which have similar phenotype to bone marrow CD138^+^ cells. The large amounts of plasma cells that can be generated with this system can now be exploited to produce differentiated B cells from patients or vaccinated volunteers, enabling the further characterization of memory B cells specific to a known antigen. Moreover, ex vivo production of plasma cells could also, as proposed elsewhere [28], be used for their protective role during stem cell transplantations in immunocompromised patients. The cells generated in the system described herein could thus become a new source of passive immunity during the period of vulnerability to opportunistic infections before the complete restoration of the patients' immune system.

## Supplementary Material

Supplemental Material included a diagram showing the culture experimental model and ELISPOTS representative of the Ig-secreting patterns of B cells submitted to interaction with CD154 and CD70.

## Figures and Tables

**Table 1:** CD31^+^ and CD39^+^ cell frequencies within plasma cells in vitro and in vivo.

	Phenotype of CD38^hi^ CD138^+/−^ cells^3^					
Cell origin^1^	Subsets^2^		CD39		CD31	
	CD138	(%)	MFI	(%)	MFI	(%)
CD70	neg	23 ± 3	515 ± 127	81 ± 6	180 ± 19	29 ± 7
	+	21 ± 4	687 ± 78	91 ± 2	568 ± 108	77 ± 2

CD154	neg	26 ± 3	512 ± 100	81 ± 6	140 ± 23	25 ± 7
	+	18 ± 2	694 ± 69	91 ± 2	526 ± 72	74 ± 3

Blood	neg	0.16 ± 0.02	44 ± 14	34 ± 5	310 ± 44	81 ± 5
	+	0.15 ± 0.03	59 ± 11	34 ± 5	1547 ± 223	93 ± 2

Bone marrow	neg	2.17 ± 0.28	9 ± 0.3	34 ± 2	67 ± 7	43 ± 2
	+	0.41 ± 0.08	52 ± 2.8	54 ± 2	194 ± 12	80 ± 3

^1^B lymphocytes following in vitro differentiation in the presence of CD70 (*n* = 10) or CD154 (*n* = 13) for 11 and 14 days or mononuclear cells isolated from peripheral blood (*n* = 14) and bone marrow samples (*n* = 10).

^2^The frequency of CD38^hi^ cells was further analyzed in relation to the expression of CD138.

^3^CD31 and CD39 frequency and MFI were determined on CD38^hi^CD138^−^ (neg) and CD38^hi^CD138^+^ (+) as indicated.

## References

[1] Oracki S. A., Walker J. A., Hibbs M. L., Corcoran L. M., Tarlinton D. M. (2010). Plasma cell development and survival. *Immunological Reviews*.

[2] Shapiro-Shelef M., Calame K. C. (2005). Regulation of plasma-cell development. *Nature Reviews Immunology*.

[3] Tarlinton D., Radbruch A., Hiepe F., Dörner T. (2008). Plasma cell differentiation and survival. *Current Opinion in Immunology*.

[4] Lacotte S., Decossas M., Le Coz C., Brun S., Muller S., Dumortier H. (2013). Early differentiated CD138^high^ MHCII^+^ IgG^+^ plasma cells express CXCR3 and localize into inflamed kidneys of lupus mice. *PLoS ONE*.

[5] Kawano M. M., Mihara K., Huang N., Tsujimoto T., Kuramoto A. (1995). Differentiation of early plasma cells on bone marrow stromal cells requires interleukin-6 for escaping from apoptosis. *Blood*.

[6] Wang L. D., Wagers A. J. (2011). Dynamic niches in the origination and differentiation of haematopoietic stem cells. *Nature Reviews Molecular Cell Biology*.

[7] Manz R. A., Hauser A. E., Hiepe F., Radbruch A. (2005). Maintenance of serum antibody levels. *Annual Review of Immunology*.

[8] Jego G., Palucka A. K., Blanck J.-P., Chalouni C., Pascual V., Banchereau J. (2003). Plasmacytoid dendritic cells induce plasma cell differentiation through type I interferon and interleukin 6. *Immunity*.

[9] Calame K. L., Lin K.-I., Tunyaplin C. (2003). Regulatory mechanisms that determine the development and function of plasma cells. *Annual Review of Immunology*.

[10] Berglund L. J., Avery D. T., Ma C. S. (2013). IL-21 signalling via STAT3 primes human naive B cells to respond to IL-2 to enhance their differentiation into plasmablasts. *Blood*.

[11] Deenick E. K., Avery D. T., Chan A. (2013). Naive and memory human B cells have distinct requirements for STAT3 activation to differentiate into antibody-secreting plasma cells. *The Journal of Experimental Medicine*.

[12] Rodríguez-Bayona B., Ramos-Amaya A., López-Blanco R., Campos-Caro A., Brieva J. A. (2013). STAT-3 activation by differential cytokines is critical for human in vivo-generated plasma cell survival and Ig secretion. *The Journal of Immunology*.

[13] Néron S., Nadeau P. J., Darveau A., Leblanc J.-F. (2011). Tuning of CD40-CD154 interactions in human B-lymphocyte activation: a broad array of in vitro models for a complex in vivo situation. *Archivum Immunologiae et Therapiae Experimentalis*.

[14] Banchereau J., Bazan F., Blanchard D. (1994). The CD40 antigen and its ligand. *Annual Review of Immunology*.

[15] Néron S., Racine C., Roy A., Guérin M. (2005). Differential responses of human B-lymphocyte subpopulations to graded levels of CD40-CD154 interaction. *Immunology*.

[16] Fecteau J. F., Roy A., Néron S. (2009). Peripheral blood CD27^+^ IgG^+^ B cells rapidly proliferate and differentiate into immunoglobulin-secreting cells after exposure to low CD154 interaction. *Immunology*.

[17] Takubo K., Goda N., Yamada W. (2010). Regulation of the HIF-1*α* level is essential for hematopoietic stem cells. *Cell Stem Cell*.

[18] Nolte M. A., van Olffen R. W., van Gisbergen K. P. J. M., van Lier R. A. W. (2009). Timing and tuning of CD27-CD70 interactions: the impact of signal strength in setting the balance between adaptive responses and immunopathology. *Immunological Reviews*.

[19] Borst J., Hendriks J., Xiao Y. (2005). CD27 and CD70 in T cell and B cell activation. *Current Opinion in Immunology*.

[20] Agematsu K., Hokibara S., Nagumo H., Shinozaki K., Yamada S., Komiyama A. (1999). Plasma cell generation from B-lymphocytes via CD27/CD70 interaction. *Leukemia and Lymphoma*.

[21] Kobata T., Jacquot S., Kozlowski S., Agematsu K., Schlossman S. F., Morimoto C. (1995). CD27-CD70 interactions regulate B-cell activation by T cells. *Proceedings of the National Academy of Sciences of the United States of America*.

[22] Nagumo H., Agematsu K. (1998). Synergistic augmentative effect of interleukin-10 and CD27/CD70 interactions on B-cell immunoglobulin synthesis. *Immunology*.

[23] Morimoto S., Kanno Y., Tanaka Y. (2000). CD134L engagement enhances human B cell Ig production: CD154/CD40, CD70/CD27, and CD134/CD134L interactions coordinately regulate T cell-dependent B cell responses. *The Journal of Immunology*.

[24] Auner H. W., Beham-Schmid C., Dillon N., Sabbattini P. (2010). The life span of short-lived plasma cells is partly determined by a block on activation of apoptotic caspases acting in combination with endoplasmic reticulum stress. *Blood*.

[25] Pelletier N., Casamayor-Pallejà M., de Luca K. (2006). The endoplasmic reticulum is a key component of the plasma cell death pathway. *Journal of Immunology*.

[26] Chu V., Beller A., Nguyen T. T., Steinhauser G., Berek C. (2011). The long-term survival of plasma cells. *Scandinavian Journal of Immunology*.

[27] Amanna I. J., Slifka M. K. (2010). Mechanisms that determine plasma cell lifespan and the duration of humoral immunity. *Immunological Reviews*.

[28] Caraux A., Klein B., Paiva B. (2010). Circulating human b and plasma cells. age-associated changes in counts and detailed characterization of circulating normal CD138^−^ and CD138^+^ plasma cells. *Haematologica*.

[29] Medina F., Segundo C., Campos-Caro A., González-García I., Brieva J. A. (2002). The heterogeneity shown by human plasma cells from tonsil, blood, and bone marrow reveals graded stages of increasing maturity, but local profiles of adhesion molecule expression. *Blood*.

[30] Tarte K., De Vos J., Thykjaer T. (2002). Generation of polyclonal plasmablasts from peripheral blood B cells: a normal counterpart of malignant plasmablasts. *Blood*.

[31] Avery D. T., Ellyard J. I., Mackay F., Corcoran L. M., Hodgkin P. D., Tangye S. G. (2005). Increased expression of CD27 on activated human memory B cells correlates with their commitment to the plasma cell lineage. *The Journal of Immunology*.

[32] Cocco M., Stephenson S., Care M. A. (2012). In vitro generation of long-lived human plasma cells. *Journal of Immunology*.

[33] Jourdan M., Caraux A., De Vos J. (2009). An in vitro model of differentiation of memory B cells into plasmablasts and plasma cells including detailed phenotypic and molecular characterization. *Blood*.

[34] Huggins J., Pellegrin T., Felgar R. E. (2007). CpG DNA activation and plasma-cell differentiation of CD27^−^ naive human B cells. *Blood*.

[35] De Vos J., Hose D., Rème T. (2006). Microarray-based understanding of normal and malignant plasma cells. *Immunological Reviews*.

[36] Elgueta R., de Vries V. C., Noelle R. J. (2010). The immortality of humoral immunity. *Immunological Reviews*.

[37] Simsek T., Kocabas F., Zheng J. (2010). The distinct metabolic profile of hematopoietic stem cells reflects their location in a hypoxic niche. *Cell Stem Cell*.

[38] Joo H., Coquery C., Xue Y. (2012). Serum from patients with SLE instructs monocytes to promote IgG and IgA plasmablast differentiation. *The Journal of Experimental Medicine*.

[39] Suyani E., Sucak G. T., Akyürek N. (2013). Tumor-associated macrophages as a prognostic parameter in multiple myeloma. *Annals of Hematology*.

[40] Gelfand E. W., Ochs H. D., Shearer W. T. (2013). Controversies in IgG replacement therapy in patients with antibody deficiency diseases. *Journal of Allergy and Clinical Immunology*.

[41] Hiepe F., Dörner T., Hauser A. E., Hoyer B. F., Mei H., Radbruch A. (2011). Long-lived autoreactive plasma cells drive persistent autoimmune inflammation. *Nature Reviews Rheumatology*.

[42] Néron S., Roy A., Dumont N. (2012). Large-scale in vitro expansion of polyclonal human switched-memory B lymphocytes. *PLoS ONE*.

[43] Néron S., Thibault L., Dussault N. (2007). Characterization of mononuclear cells remaining in the leukoreduction system chambers of apheresis instruments after routine platelet collection: a new source of viable human blood cells. *Transfusion*.

[44] Néron S., Pelletier A., Chevrier M.-C., Monier G., Lemieux R., Darveau A. (1996). Induction of LFA-1 independent human B cell proliferation and differentiation by binding of CD40 with its ligand. *Immunological Investigations*.

[45] Néron S., Suck G., Ma X.-Z. (2006). B cell proliferation following CD40 stimulation results in the expression and activation of Src protein tyrosine kinase. *International Immunology*.

[46] Fecteau J. F., Néron S. (2003). CD40 stimulation of human peripheral B lymphocytes: distinct response from naïve and memory cells. *The Journal of Immunology*.

[47] Radbruch A., Muehlinghaus G., Luger E. O. (2006). Competence and competition: the challenge of becoming a long-lived plasma cell. *Nature Reviews Immunology*.

[48] Moore K. W., de Waal Malefyt R., Coffman R. L., O'Garra A. (2001). Interleukin-10 and the interleukin-10 receptor. *Annual Review of Immunology*.

[49] Jacquot S., Kobata T., Iwata S., Morimoto C., Schlossman S. F. (1997). CD154/CD40 and CD70/CD27 interactions have different and sequential functions in T cell-dependent B cell responses: enhancement of plasma cell differentiation by CD27 signaling. *The Journal of Immunology*.

[50] Maecker H. T., McCoy J. P., Nussenblatt R. (2012). Standardizing immunophenotyping for the human immunology project. *Nature Reviews Immunology*.

[51] Van De Veen W., Stanic B., Yaman G. (2013). IgG4 production is confined to human IL-10-producing regulatory B cells that suppress antigen-specific immune responses. *The Journal of Allergy and Clinical Immunology*.

[52] Deaglio S., Aydin S., Grand M. M. (2010). CD38/CD31 interactions activate genetic pathways leading to proliferation and migration in chronic lymphocytic leukemia cells. *Molecular Medicine*.

[53] Omana-Zapata I., Oreizy F., Mosqueda F. (2013). Performance of a novel BD stem cell enumeration kit on two flow cytometry systems. *International Journal of Laboratory Hematology*.

[54] Vestrheim A. C., Moen A., Egge-Jacobsen W., Bratlie D. B., Michaelsen T. E. (2013). Different glycosylation pattern of human IgG1 and IgG3 antibodies isolated from transiently as well as permanently transfected cell lines. *Scandinavian Journal of Immunology*.

[55] Shima T., Forraz N., Sato N. (2013). A novel filtration method for cord blood processing using a polyester fabric filter. *International Journal of Laboratory Hematology*.

[56] Mainou-Fowler T., Porteous A., Nicolle A., Proctor S. J., Anderson J. J., Summerfiled G. (2008). CD31 density is a novel risk factor for patients with B-cell chronic lymphocytic leukaemia. *International Journal of Oncology*.

[57] Chu V. T., Berek C. (2013). The establishment of the plasma cell survival niche in the bone marrow. *Immunological Reviews*.

[58] Medina F., Segundo C., Brieva J. A. (2000). Purification of human tonsil plasma cells: pre-enrichment step by immunomagnetic selection of CD31^+^ cells. *Cytometry*.

[59] Hong S., Lee H. W., Chang D. Y. (2013). Antibody-secreting cells with a phenotype of Ki-67^low^, CD138^high^, CD31^high^, and CD38^high^ secrete nonspecific IgM during primary hepatitis A virus infection. *Journal of Immunology*.

[60] Lanio N., Sarmiento E., Gallego A. (2013). Alterations of naïve and memory B-cell subsets are associated with risk of rejection and infection in heart recipients. *Transplant International*.

[61] González-García I., Ocaña E., Jiménez-Gómez G., Campos-Caro A., Brieva J. A. (2006). Immunization-induced perturbation of human blood plasma cell pool: progressive maturation, IL-6 responsiveness, and high PRDI-BF1/BLIMP1 expression are critical distinctions between antigen-specific and nonspecific plasma cells. *The Journal of Immunology*.

[62] Deaglio S., Mallone R., Baj G. (2000). CD38/CD31, a receptor/ligand system ruling adhesion and signaling in human leukocytes. *Chemical Immunology*.

