# Supplementary material for: Human CD38hiCD138+ Plasma Cells Can Be Generated In Vitro from CD40-Activated Switched-Memory B Lymphocytes
Source: J Immunol Res. 2014 Dec 23;2014:635108. doi: 10.1155/2014/635108 (PMC4352507; doi:10.1155/2014/635108)
Supplement: Supplementary file 1 — Supplemental Material included a diagram showing the culture experimental model and ELISPOTS representative of the Ig-secreting patterns of B cells submitted to interaction with CD154 and CD70. [file 635108.f1.docx]

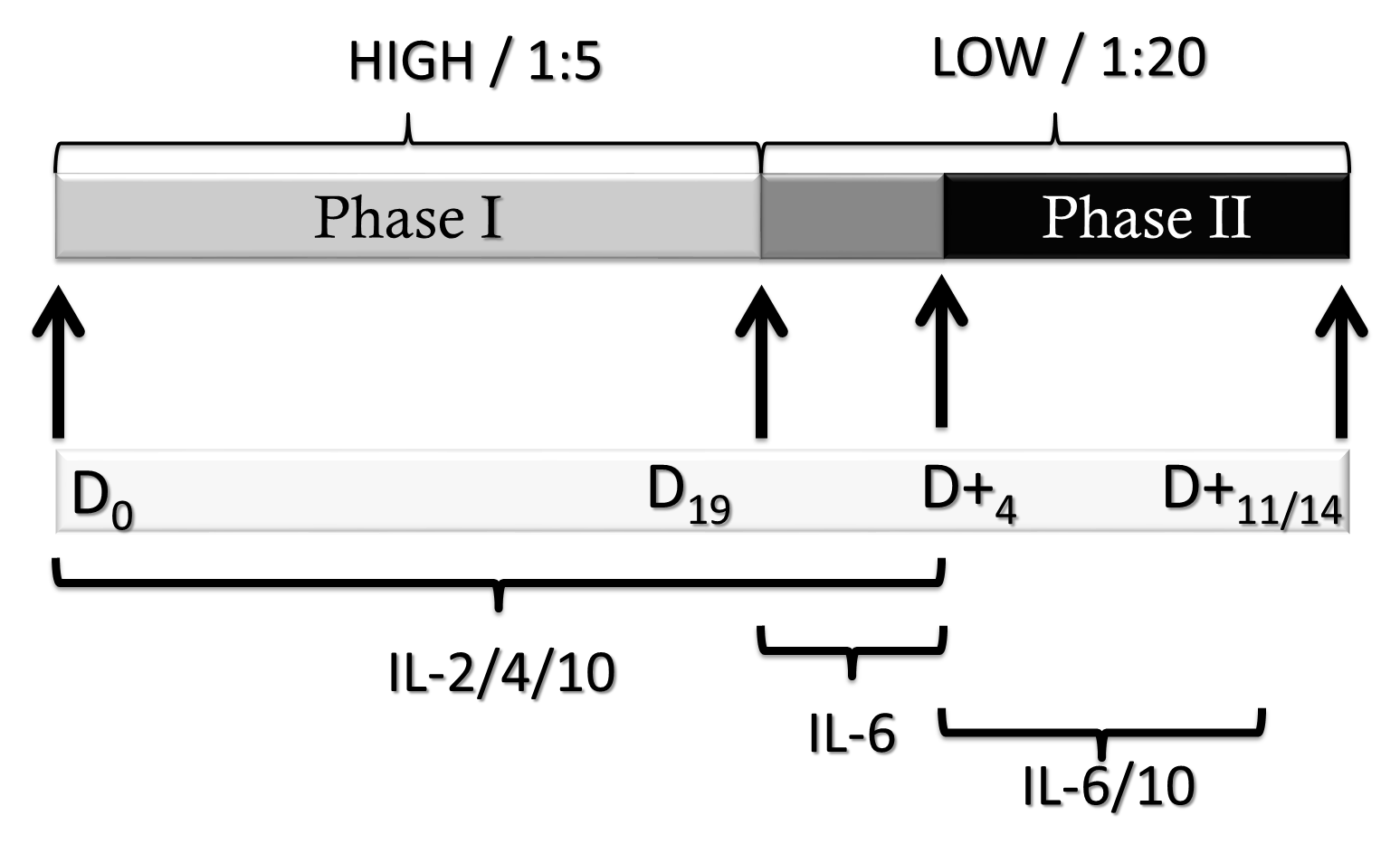
Supplemental Figure 1: Experimental model

Purified switched memory B lymphocytes were cultured for 19 days in the presence of a high level of CD154-CD40 interaction (1 :5 ratio) provided by the contact with L4.5 cell line (Phase I or expansion phase). The cells were then cryopreserved or not. As a second step (Transition between Phase I and Phase II) the expanded cells (fresh or thawed) were transferred in the presence of a low level of interaction (1:20 ratio) provided by the L4.5 cell line (CD154+ cells) or by the 3H7 cell line (CD70+ cells). The cytokines were as indicated a mix of IL-2, IL-4 and IL-10 during Phase I, then IL-6 was added for the transition and IL-2 and IL-4 were removed and replace by IL-10. The culture days are indicated for each step, namely 19 days for Phase I, 4 days of transition (D +4) and 7 to 10 supplemental days (D + 11/14).
