# Supplementary material for: Human CD38hiCD138+ Plasma Cells Can Be Generated In Vitro from CD40-Activated Switched-Memory B Lymphocytes
Source: J Immunol Res. 2014 Dec 23;2014:635108. doi: 10.1155/2014/635108 (PMC4352507; doi:10.1155/2014/635108)
Supplement: Supplementary file 2 [file 635108.f2.docx]

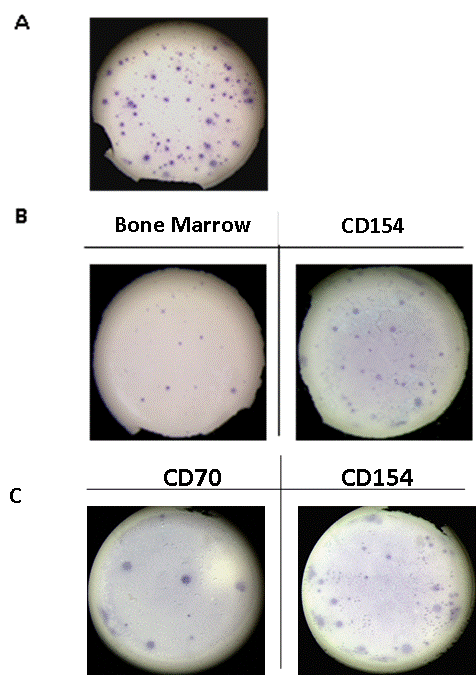


**Supplemental Figure 2: Secreting patterns of bone marrow PC compared to in vitro generated PC.**

**(**A) ELISPOT assays were done to determine the secretion patterns of bone marrow plasma cells; in this example about 0.13% of total seeded cells were IgG secreting cells. (B) B lymphocytes were stimulated for 9 days in differentiation conditions and compared to bone marrow. Each patterns presented here contained 62 spots; corresponding to 62,500 seeded bone marrow cells (0.09%) and 625 differentiated B lymphocytes (10%). (C) B lymphocytes stimulated for 9 days in differentiation conditions using CD70 or CD154 feeder cells were compared for their IgG-secreting cells frequencies. In this example, CD70 and CD154 interaction gave 16% and 31% of secreting cells, respectively. Bone marrow ELISPOT patterns are representative of 5 independent assays while ELISPOT patterns for B lymphocytes submitted to either CD154 or CD70 interaction are representative of three independent assays.
